# Pemphigus1Read before the Buffalo Academy of Medicine, February 12, 1901.

**Published:** 1902-04

**Authors:** J. W. Grosvenor

**Affiliations:** Buffalo, N. Y.


### Pemphigus.[1]

By J. W. GROSVENOR, M. D., Buffalo, N. Y.

THE word pemphigus, derived from the Greek pemphix, signifies a bladder or blister. The disease pemphigus has for synonyms pompholix, febris bullosa, phlyctena, bulla, febris vesicularis, hydatis. It is characterised by the formation of blebs or blisters, oval, or round in form, on the external surface

---

[1] Read before the Buffalo Academy of Medicine, February 12, 1901.

of the body and mucous membranes, appearing singly or in groups. The blebs contain a fluid which, on their bursting, dries into scabs and crusts, at first yellowish and then brownish in color.

Dermatologists have divided cases of this disease into various classes, each class being named according to some peculiarity of the cases placed under it. Martin, in his work published in 1829, enumerated 97 forms of the disease. Some of the more prominent of these classes are as follows, viz.: P. gangrenosus, in which there is a sloughing and gangrenous condition at the base of the blebs; P. diutinus, a very chronic form in which there appears daily, or every few days, a new crop of bullæ; P. solitarius, a form in which a single bulla appears at one time, to be followed on its disappearance by another; P. foliaceus, a form in which the bullæ leave behind large scales; P. pruriginosus, a form accompanied by intense itching; P. hemorrhagicus, a form in which a bloody exudation occurs in the bullæ; P. vegetans, a form in which the site of the bullæ becomes covered with vegetating masses, fungoid in appearance.

All the cases suitably arranged under these or other classes which have been made, may be properly divided into two classes, acute and chronic. By some observers the existence of acute pemphigus is denied. It is said to be extremely rare in adults.

Hebra,[1] the celebrated dermatologist of Vienna, denied its existence, as did also Willan and Bateman.[2] Henoch[3] believed that as a specific disease acute pemphigus did not exist, that the condition socalled is due to certain deleterious products in the blood. Of late years too many cases of acute pemphigus, both in adults and children, have been noted and described by careful and experienced investigators to reasonably doubt its existence.

Hyde[4] does not believe in such a disease as pemphigus. He regards it as a symptom of various diseases. Although a pemphigoid eruption occurs in several diseases and under many different conditions, it is true that pemphigus has distinctive characteristics which constitute it not merely a symptom but a disease. It presents a distinct clinical picture which does not represent any other disease. Blebs or blisters or bullæ appear as a concomitant of eczema, ecthyma, rupia, impetigo, urticaria, scabies, as a result of insect wounds, of certain medicines, taken internally, of the external application of some medicines, but in all such cases persistent inquiry will disclose the origin of the bullous eruption.

The blebs of pemphigus vary in size from a pea to half a hen's egg and sometimes cover an even larger area. They contain a fluid which is generally serous, sometimes purulent, sometimes sanguinolent. They may appear upon any portion of the body, but are seldom seen on the face, palms of hands and soles of feet. Sometimes they are scattered over the whole external surface and are so thickly crowded together as almost to completely envelope the body. Sometimes only a few are present simultaneously, and in certain cases only a solitary bleb can be found. In some cases so rapidly are they developed, that a score or two scores or more new ones will make their appearance in a single night.

The chronic form of the disease is found almost exclusively in adults. Children seldom suffer from chronic pemphigus. The chronicity varies in duration from a few months to a score or more of years. Addison[5], of Guy's hospital, reports the case of a girl 17 years of age, who had suffered from the disease since birth. A case is reported by Beevor[6] of a female 25 years of age, who had been the subject of the disease since two weeks old. A male patient in M. Biett's[7] ward, 30 years of age, had been affected with pemphigus from infancy.

The ordinary chronic pemphigus, frequently termed P. vulgaris, is generally observed in old people who have become debilitated. De Ricci[8] cites a case in a male over 80 years of age. Sometimes it begins with a slight febrile disturbance and sometimes without any excess of temperature.

Usually there is a feeling of malaise, and within a few hours or a day or two on some part of the external surface of the body red points appear on which blebs arise, sometimes few, sometimes many. They may or may not be surrounded by an areola. A new crop may appear each day or after the intervention of several days. A slightly feverish condition is apt to occur at the advent of each successive crop. The blebs, dry in a day or two and leave behind upon an ulcerated base a scab or crust which falls off in a few days, leaving a dark-colored pigmentation. Not infrequently the eruption is accompanied by burning and itching sensations which are almost intolerable, compel the patient to be divested of all clothing and take to the bed. At times the eruption appears in the mouth, on the palate and pharynx, in the vagina, on the conjunctiva and mucous membrane in other parts of the body. An interesting and unusual case of chronic P. vulgaris of the mouth and epiglottis has been reported by Dr. Lewis H. Miller[9]. The patient was a male, 72 years of age. The eruption affected only the mouth and epiglottis for over twenty months.

Gasetti[10] found on record twenty cases in which the conjunctiva was affected and in every case complete and irreparable blindness occurred by transformation of cornea and conjunctiva into cicatricial tissue. Lang[11] has reported two cases of conjunctival pemphigus; one was the case of a woman 52 years of age; in the other case the eruption was present on other parts of the body and could be produced by a vigorous shake of the hand.

This symptomatic picture of chronic pemphigus may continue for many years, the disease at times almost entirely disappearing and suddenly breaking out with renewed violence. Sherwell[12] reports a case of P. foliaceus, which relapsed eleven years after recovery. It recovered from the relapse.

The patient may die from exhaustion caused by the disease or from some intercurrent affection.

The form of chronic pemphigus termed P. vegetans, was first recognised and described by Neumann[13]. Hyde[14] was the first to report a case in the United States. This form is exceedingly rare and but few cases have been recorded. After the rupture of the bullæ, which at first resemble those of other forms of pemphigus, fungous growths appear on their site, rise above the surrounding surface and secrete a muco-purulent fluid which is very offensive. Crocker[15] reports a typical case of this description in a woman 43 years of age. She died after three months. The bullæ appeared not only on the external surface but on the oral mucosa, conjunctiva and vulva where there was extensive denudation. In some cases suppurative onychitis of fingers and toes has occurred resulting in a shedding of the nails. In such severe cases the disease is uniformly fatal.

P. foliaceus is another chronic form of the disease. This form is characterised by the production of large scabs or squamæ from the bullæ. The blebs appear in successive crops and become confluent. Oftentimes there issues from them an offensive odor. These scales vary in size from ¾ of an inch to 2 inches in diameter. It is an extremely rare form of pemphigus. Flaccidity is characteristic of the bullæ. They are not distended nor tense. Often they are so ill-defined that the epidermis is hardly raised from the tissue beneath it. Their contents, at first clear or lactescent, become purulent or sanguinolent. The crusts which form after the rupture of the bullæ are attached by their center to the underlying tissue while their edges remain free. The eruption spreads until it covers the body. The mouth and throat are affected. Contractions on the face may produce ectropion, followed by conjunctivitis. Ulcerated surfaces are deep. Palms and soles become infiltrated and fissured. Nails become distorted and are shed. Hair of scalp and beard falls out. There are sensations of burning, smarting and soreness. This form of pemphigus is said to be more frequent in women than in men. It is fatal in a large majority of cases.

Munro[16] and Swarts report a case of P. foliaceus malignus in a woman 37 years of age. The blebs finally spread over the whole body and presented a wrinkled and flaccid appearance. Although the progress of the case was regarded as favorable the patient died from exhaustion after eighteen weeks of treatment.

The acute form of pemphigus occurs almost exclusively in children, and hence has been called P. neonatorum. Indeed, some authors declare that the acute form is never seen in adults. Well-marked acute cases in adults have been reported by several eminent dermatologists. It is generally acknowledged, however, that such cases are rare. M. Haroud[17] reported two acute cases in the Lyon Medical of August 3, 1873; one in a young man 17 years of age, and the other in a girl 14 years of age. Izett W. Anderson[18] saw an acute case in a male adult negro which followed the eating of a semi-putrid conger eel. Duckworth[19] reports a case in a railway guard 54 years of age. Death took place in nine days. Allen[20] reports a case in a man 33 years of age which lasted a month. Sparks, Payne and Southey[21] have recorded cases of it. Erik Pontoppidan[22] states that he saw 20 cases in adults in a single year.

The children in whom it most frequently occurs are ill-nourished and cachectic. It sometimes occurs, however, in children who are healthy. Cases of the latter class almost uniformly have a favorable termination.

In the severe cases of children there is an ichorous discharge from the base of the bullæ, followed by sloughing and gangrene. Such cases are often styled P. gangrenosus, and are developed in the first three years of life in children who are poor and live in moist localities. Sometimes the gangrenous form destroys the external ear and extends into the auditory canal, sometimes into the eyes and destroys the sight.

The acute form of the disease in children may begin with or without febrile symptoms. The blebs sometimes form before birth, sometimes appear within a fortnight after birth. Any part of the body may be affected, but the lower parts of the trunk, which are apt to be uncleanly, are most likely to suffer. The face, hands and feet are often free from the eruption. The conjunctiva may be affected and also the buccal mucosa.

In some instances the disease is hemorrhagic. In favorable cases it completes its course in three or four weeks. The fatal cases occur in children who are ill-nourished, live in an unhealthy environment and are subjected to some kind of infection.

In adults the acute form may be marked by febrile disturbance the same as in children. The bullous eruption may be sparse or abundant, in some cases covering the entire body and attacking the mucous surfaces.

A diphtheritic exudation occasionally is found on the ulcerated surface under the bullæ. Bulkley[23] has described a case of herpes gestationis which is said by Weyl to be an example of acute P. adultorum.

The rarity of pemphigus has been shown by statistics from various sources. According to statistics of the American Dermatological Association[24] there were 14 cases of pemphigus out of 16,863 cases of skin diseases. White[25], of Boston, reports that he met 15 cases of pemphigus in 5,000 cases of skin disease. McCall Anderson[26] states that out of 24,891 cases of skin disease only 53 were pemphigus. Bulkley[27] reports 17 cases of pemphigus out of 8,000 cases of skin disease in this country. In 7,000 cases of sick children in Bellevue Hospital there were only two cases of pemphigus.[28] The American Dermatological Association[29] has reported that 30 cases of pemphigus occurred in the United States and Canada in 1894.[30] In General Hospital, Lying-in Hospital and Foundling Hospital of Vienna, there occurred annually one case of pemphigus in every 10,000 adults and one in every 700 infants at the breast. Hebra[31] states that he had seen 50 cases of pemphigus at his clinic. He believed that the disease appeared 14 times as frequently in infants as in adults.

*Etiology.*—Concerning the etiological factors of pemphigus a variety of opinions has been recorded. The discovery of the *basal* cause may be regarded as quite doubtful. Sex does not seem to exert a marked influence as a causal factor. Duhring[32] states that both sexes suffer from it in the same proportion.

Robinson[33] states that P. foliaceus is more frequent in women than in men; he also states that P. vulgaris occurs equally in both sexes. Hebra thinks that the disease is about equally divided between the sexes.

Hardy[34] has described a form of pemphigus which he has seen in females from 14 to 20 years of age, who were unmarried and were suffering from irregular menstruation. Others have mentioned a P. hystericus occurring in hysterical females of the same class. Duncan[35] mentions a case of recurrent P. pruriginosus which occurred in an unmarried woman 25 years of age, immediately after the checking of the menses at two successive periods.

Hardy[36] and Wilson have observed cases of P. pruriginosus in pregnancy.

In Wilson's case it occurred in every pregnancy. Cases are related by Barnes[37] in which pemphigus occurred during several successive pregnancies. It is related of a woman 37 years of age that she suffered from pemphigus after four of 12 of her confinements.

Cases are cited in which bullæ appear after the ingestion of certain medicines, as iodide potassium, Donovan's solution, Fowler's solution.

In a case of acute pemphigus of the hands which came under Startin's[38] care the patient, a man 32 years of age, had taken Fowler's solution for three months and followed it with Donovan's solution. On omitting both medicines he rapidly improved.

Style[39] believes that two cases of acute pemphigus which he saw in children, respectively five and two years of age resulted from the inhalation of sewer gas.

Owen[40] relates the case of a girl 10 years of age who had a good family history. After a very hot day and drinking a large quantity of water the soles of the feet were studded with blisters. Blebs also appeared on palms of hands.

Some writers hold the opinion that infantile pemphigus is syphilitic in its origin and others are opposed to this view. It is probable that pemphigus is a complication of some cases of infantile syphilis. It is also probable that some cases of infantile pemphigus have no relation to syphilis. Staub[41] believes that pemphigus of the new-born is most frequently the result of a puerperal infection in the mother. He cites two cases in which the mother had a severe pyemic puerperal fever accompanied by a pemphigoid eruption; the child had a typical P. neonatorum.

The questions: Is pemphigus epidemic? Is pemphigus contagious? have received contradictory answers from different observers. The form P. gangrenosus of children has prevailed as an epidemic in Ireland[42]. Nelligan[43] believes that the chronic form appears as an epidemic. Ziemssen[44] says that the form which occurs in children is sometimes epidemic. He thinks it undoubtedly contagious. Scharlan[45] in 1841 reported an epidemic in a family. In Hospice des Enfants Assistis[46] there occurred an epidemic of sixteen cases in children whose ages varied from two days to thirteen months. Behrend[47] believes that in the new-born the disease occurs almost exclusively as an epidemic, so that the theory of contagion in such cases is warranted. Padora[48] reports an epidemic which occurred in Pavia. Fomolle[49] reports an epidemic in la Charite, lasting two and a half months. During this time there were 77 births and but few of the children escaped the disease. Contagion by direct contact seemed evident in one case. A similar epidemic occurred in the Lying-in Hospital in Leipzig.[50] Ballard[51] reports a case in which pemphigus was communicated from a cow to a human being. Beyerlein[52] believes the disease contagious and generally epidemic when it affects not only infants but older children and this infantile form is sometimes transferred to adults.

Salvage[53] reports the case of a nursing child on whom bullæ were observed three days after birth. Fresh bullæ appeared daily for fifteen days. Four days before bullæ ceased to be formed on the child several bullæ were observed on the breasts and forearms of the mother. Apparently the mother's case was the result of contagion.

Sturges[54] reports the case of a male child on whose fingers, six days after birth, bullæ appeared, followed in three or four days by bullæ on the face. Afterwards bullæ appeared on four or five other children of the same family, on the father and on the nurse, who attended the mother.

Lattey[55] reports 4 cases in children in which the disease was probably contagious; one aged 2½ years, one 7 years, one 7 months. The fourth, who was 9 years old, was the first attacked and became convalescent before the eruption appeared on the others. An epidemic is said by Tilbury Fox[56] to have taken place in the General Lying-in Hospital in London, England. He further states that in its epidemic form the disease "appears to be the result of acute blood poisoning." Hebra[57] did not believe in the contagiousness of pemphigus. He inoculated himself without effect. Neither Duhring nor Robinson[58] believe in its contagion.

The reports of exact observers to the effect that the pemphigus of children is epidemic and contagious are too numerous to permit us to doubt that contagion is an etiological factor of the disease.

Henoch[59] states that he never saw a case in which infection could be proved. Shillitoe[60] describes a case in a girl 19 years of age which, on bacteriological examination showed a diplococcus in the fluid of the bullæ at one stage of the disease and at another stage, streptococci and staphylococci.

L. Emmett Holt[61] reports a case of P. neonatorum associated with a general infection by the staphylocccus pyogenes. The infant was 9 days old when admitted to the hospital. No history of syphilis in the parents and other two children could be elicited. Bullæ from ¼ inch to 2 inches in diameter spread over various parts of the body. Cultures from one bulla on the thigh and another on the neck showed staphylococcus pyogenes aureus. Pus organisms were found in pus from the eyes but no gonococci.

Cultures from the lungs showed staphylococcus pyogenes aureus and bacterium lactis ærogenes; from the spleen and left kidney, staphylococcus longus; from the liver, streptococcus longus and staphylococcus pyogenes aureus. Cultures of staphylococcus pyogenes aureus were injected into a mouse which was found dead in 24 hours. This organism was obtained from the heart's blood of the mouse and the site of injection. The child died 60 hours after admission to the hospital. The general infection in this case is quite noticeable.

The question of heredity of pemphigus has occupied the attention of a few observers. Robinson[62] states that in some cases it seems to be hereditary. An inherited form has been described by Goldschneider, Legg and others.[63] Kaposi[64] relates an inherited case in which patient's mother, sister, mother's brother and some of latter's children were affected with the disease.

According to Vierordt,[65] pemphigus sometimes rests on a neurotic basis and may be regarded as a disease of the peripheral nerves. Hyde[66] asserts that sufficient proof has been adduced to warrant the belief that changes in the trophic nerves and nervous centers are causes of the disease. Lesions of the spinal cord have been followed by a bullous eruption. It is true, however, that in such cases the blebs do not resemble in all respects those which occur from other forms of pemphigus. It is also true that in fatal cases of pemphigus the changes in the spinal cord are not uniform in their site and appearance. Kaposi[67] and Weiss by autopsy of 9 bodies dead from pemphigus found changes in the cord in only 1, diffuse sclerosis. Is it not quite probable that the cutaneous and nervous lesions of this disease are due to toxic agents?

Järisch[68] found swellings of the processes of ganglion cells and interstitial fibrous deposits. Déjerine and Leloir[69] found in peripheral nerves changes due to degeneration. In 1882, Gibier[70] found bacteria in the contents of bullæ of acute pemphigus. He states that the microbes when mature were arranged in chaplets (a row of links in form of a rosary) each containing a series of joints. His observations were confirmed by Vidal and Roeser.[71] In 1883, Riehl[72] found both conidia and spores in the epidermic layer underneath the blebs of infantile pemphigus. In 1886, Demme[73] found cocci in the contents of the bullæ and in the blood. Thin,[74] of London, attempted to discover microörganisms in the contents of bullæ by different methods of staining and cultivation with negative results.

M. Déjerine[75] reported to the Paris Academy of Sciences a case of general paralysis. An eruption of bullæ appeared on the legs and arms. An examination after death showed sclerosis of lateral columns of spinal cord. Gray matter and posterior columns were healthy. By microscopical examination of skin and areolar tissue removed from the site of bullæ, "nerve tubes were found altered in structure and no trace of axis cylinder was discoverable." Déjerine[76] held the opinion that "the alteration of the nerve fibers extended from these peripheral ends to the trophic centers whereon the nutrition, as well of the cutaneous nerves as of the skin itself is dependent."

Vidal[77] has demonstrated that adults can be successfully inoculated from the contents of the bullæ.

Owen[78] believes that pemphigus is due to some cause acting through the nervous system, though exact cause is obscure.

Ziemssen[79] states that the inoculated secretion of the bullæ in from 24 to 72 hours shows a formation of vesicles attended by burning and redness. Roeser[80] always found micrococci in the contents of the bullæ. Tilbury Fox[81] remarks that "pemphigus would seem to be the result of vaso-motor disturbance and not of disorder of trophic nerves."

From this accumulation of recorded experiments and opinions no very positive conclusion can be drawn concerning the exact etiology of pemphigus. It is evident that certain bodily conditions are accompanied by an eruption of bullæ and a disease called pemphigus. It is extremely probable from testimony adduced that the disease is due to some microörganism which produces a degeneration of peripheral nerves and trophic centers.

It has seemed to the writer that facts in sufficiently large number have not been clearly and fully established to determine the primary cause of this disease. It may be a microbic infection. The investigations of Gibier point in that direction. It can hardly be doubted that the disease is partially due to either a functional disturbance or organic degeneration of the peripheral nerves. Back of these nerves as a causal factor may be a disorganised condition of nervous centers. It would be a rash and unwarranted declaration to state with positiveness the exact basic cause of pemphigus.

*Chemical Condition of the Contents of the Bullæ.*—Duhring[82] states that the fluid is either alkaline or neutral; the older the fluid the more alkaline it becomes. He believes that chemical analysis of blebs, urine and blood has given no insight into the cause of the disease. Neumann[83] describes the bullous fluid as clear and yellowish at first, then cloudy and purulent. It lies between the two layers of epidermis.

Nelligan[84] states that the fluid is highly albuminous. Järisch[85] found serum, pus and epithelial cells in the blebs. Tilbury Fox[86] states that the fluid generally is at first transparent, then milky, sometimes sanguinolent from the outset; at first alkaline but becomes acid.

*Diagnosis.*—The bullous eruption does not constitute the disease. An eruption of bullæ may occur in several different diseases. It is, then, in most diseases simply an accompaniment of the disease and not a necessary part of it; in such cases its appearance is accidental. The disease pemphigus never occurs without the bullous eruption which appears in successive crops and never exhibits the characteristics of other diseases. The diagnosis is easy in its fully developed stage but difficult in its beginning and later stages.

When bullæ appear in a case of syphilis a syphilitic history and antecedents can usually be elicited. The eruption in such cases is almost invariably in children and is present on soles of feet and palms of hands.

In eczema of hands the bullæ are formed by confluence of vesicles; pemphigus is rare on hands and fingers and skin is not infiltrated. In ecthyma cachecticum, the pustules are not true bullæ and ulcerations are deep. In rupia the bullæ are smaller and flatter than in pemphigus; contents are thick and dark and ulcerations deep. P. foliaceus, when the blebs become scaly, may resemble pityriasis rubra; in the latter there are no bullæ. In impetigo contagiosa the bullæ are never distended as in pemphigus but are flat. The former is a pustular disease. In bullæ of lepra there is usually cutaneous anesthesia. The blebs generally leave behind signs of atrophy on their bases. In herpes iris the eruption is rather vesicular than bullous and disappears more rapidly than the eruption of pemphigus.

Although bullæ appear in urticaria and erythema multiforme, these diseases are easily recognised by characteristic symptoms.

P. vegetans has sometimes been confounded with syphilis because the eruption in both diseases often appears about the ano-genital region and the eruption of P. vegetans resembles the condylomata of syphilis. Ulcerations in the mouth also occur in both diseases. In P. vegetans the lymphatic glands are not involved as in syphilis. The condylomata of syphilis rarely spread beyond the region adjacent to mucous outlets as does the eruption of pemphigus.

Scabies of infants and children, which sometimes is accompanied by bullæ, has a parasite which will determine the nature of the disease.

Bullæ may appear in other diseases than those mentioned, but such diseases have other symptoms which will readily distinguish them from pemphigus.

*Prognosis.*—In children the acute form generally has a favorable termination. It is apt to be fatal in children who are cachectic and ill-nourished. Children die from exhaustion, worn out from the irritative fever. The malignant form, characterised by severe and persistent fever, diphtheritic patches, ulceration, sloughing, as seen especially in children, very rarely ends in recovery. Robinson[87] regards the prognosis in children unfavorable if they are suffering from bronchial or intestinal catarrh, kidney trouble or hematuria.

In old people if the disease is general, prognosis is unfavorable.

P. gangrenosus is almost uniformly fatal. The distress occasioned by the burning and itching of P. pruriginosus is apt to lead to a fatal result. The toxic effect of the disease upon the general system may be shown by its influence on vessels and glands situated near the site of the eruption and thus septicemia may lead to a fatal ending. When the eruption affects the mouth and pharynx, mastication and deglutition may be performed with difficulty and lead to impairment of nutrition, which will invite a fatal issue. Pemphigus in adults almost always has a fatal termination, especially P. foliaceus. Hebra[88] believes that P. foliaceus invariably terminates fatally. Hyde[89] states that P. vegetans in the vast majority of cases is fatal, one or two only in about sixty cases having recovered.

The general condition of the patient should be a reliable guide in prognosis. In general it may be said that the more widely spread is the eruption, the larger and more deeply seated the bullæ, the older and more poorly nourished the patient, the greater is the probability of a fatal issue.

*Treatment.*—The treatment of pemphigus has been almost as varied as the mental inclination of the physicians who have treated it. To a large extent it has been experimental. Nearly all dermatologists have used both internal and external remedies. The treatment of the two forms, chronic and acute, should differ. The chronic form, occurring in old and enfeebled patients, demands tonic medicines and a nutritious diet.

Perhaps no one internal remedy for chronic cases has had more favorable consideration than arsenic. It is commended by Tilbury Fox.[90] Hutchinson[91] has regarded it as almost a specific in such cases. He has not met with a single case of failure in a trial of the remedy for many years. Duhring[92] considers arsenic the most valuable internal medicine in chronic pemphigus.

Allen[93] reports a case in a woman 45 years of age in which arsenic internally, and compound tincture of benzoin externally, performed a good service. Kaposi[94] reports that he could not obtain favorable results from arsenic. For P. vegetans and P. foliaceus, arsenic has been tried by different dermatologists without apparently any curative effect. Piffard's[95] treatment for P. foliaceus is arsenic in full doses. For chronic pemphigus Tilbury Fox[96] prefers quinine in full doses to arsenic. Duhring[97] highly esteems quinine as an internal remedy for chronic pemphigus, especially if the eruption is preceded by fever. Munro and Swarts[98] report a case of P. foliaceus malignus in a woman 37 years of age, cured in 14 weeks by nutritious diet, quinine, Fowler's solution, syrup iodide iron internally, and externally bichloride solution 1 to 4,000, and zinc ointment. Neumann[99] believes quinine the only internal medicine worthy of consideration in the treatment of chronic pemphigus. Eames[100] reports a case that he cured with phosphorus internally. Hebra[101] believes that no internal medicine is curative of the disease. He states, "I know of no way of curing pemphigus by giving internal medicine."

For local treatment in both acute and chronic cases a soothing and cooling powder should be used, as oxyd of zinc, starch, bismuth, calamine, lycopodium, mixed with some kind of antiseptic, as carbolic acid or boric acid; also a cooling and quieting wash, as lead and opium; also a soothing ointment, as borated oxyd of zinc. Secretan[102] reports a case of the chronic form in an adult male cured by local applications of carbolised water.

In severe chronic forms of the disease the continuous bath has been used. Kaposi[103] kept a patient in a bath day and night for eight months, which resulted in a great amelioration of the symptoms. As a local application Hebra[104] regards cold water as the best remedy. He cured some patients by use of the continuous bath, keeping them immersed continually, one, 109 days; one, 76 days; one, 47 days; one, 26 days.

In acute forms of the disease the feverish condition should be subdued, and then tonics with quinine should be used with strongly nourishing diet. Mineral acids, cod liver oil and iron, arsenic, have also been used with good effect in acute cases, both in children and adults.

In all forms of pemphigus great care should be taken to remove all impurities from the system. Doubtless in the future when the cause of the disease has been placed upon a more scientific basis, treatment will be more successful and satisfactory. The fact that pemphigus is a debilitating disease and generally attacks the old and enfeebled or the young and poorly nourished, points to a general plan of treatment which is supporting and constructive.

The following record of a case of chronic pemphigus, ordinarily termed P. vulgaris, well illustrates a typical form of the disease and presents a plan of treatment which was followed by a favorable issue.

Mrs. —— presented herself for treatment in June, 1899; 66 years of age; twice married; never been pregnant; health uniformly good; never had any skin eruption; always thin in flesh; of small stature. Inquiry could elicit no history of syphilis in her own nor the family of either husband.

Patient stated that in April, 1899, an eruption appeared on her back which was healed after the application of an ointment. On the patient's first call at the writer's office there was present on her lower extremities, mostly about the ankles, a profuse eruption, consisting of small ulcers and crusts of a dark color. No fever; bowels inclined to constipation; diminished appetite; a general appearance of not being well nourished.

The exact nature of the eruption was not diagnosticated. The treatment in the early history of the case consisted of preparations of iron and quinine and arsenic internally, and locally nitrate of mercury ointment, vaseline, simple cerate containing cocaine, witch hazel, as a cooling wash.

Late in the fall of 1899, the eruption having spread to the back, arms, thighs, body, palate, pharynx, forehead, the case having been recognised as pemphigus, and considerable anxiety being felt concerning its termination, a consultation was held with Dr. Ernest Wende, of this city, who advised pushing the arsenic to large doses and a borated and carbolised ointment of oxyd of zinc. This treatment was adopted. Arsenite of quinine was given three times daily, the dose being gradually increased from 1-50 to 1-5 of a grain. The eruption continued to spread till apparently not a square inch of the external surface of the body was free from it except soles of feet, palms of hands, face and portions of scalp. At times the itching and burning were intense.

In the early part of April, 1900, the ulcerated patches had become so numerous that the ordinary clothing could not be worn and the patient took to her bed. The bullæ came out in scores in a single night, many of them filled with a sanguinolent fluid. At this time the eruption was at its height. The arsenic preparation was stopped for a week and as a substitute 12 grains of quinine were given daily. This was followed by arsenite of potash, 3 times per day, 1-20 of a grain, the dose being gradually increased to 1-5 of a grain. The patient was confined to bed five weeks, during which time over the whole body was used a powder of talcum and boric acid, the mixture being 90 per cent. of the former and 10 per cent. of the latter. This powder in large quantities was gently rubbed upon the skin and the legs and arms enveloped in roller bandages. This local treatment was repeated every second day during the five weeks the patient was in bed and continued at longer intervals for a month thereafter. During this treatment the ulcerated surfaces were covered with a carbolised ointment of vaseline and lanolin.

During the summer and autumn of 1900 the patient was kept on an arsenic and iron treatment with occasional alternations of quinine. The borated talcum powder was used on all the eruptive surfaces.

Throughout the case a generous diet has been advocated. Early in its history it was learned that a diet of meat had been largely ignored, and hence beef and lamb in various forms were frequently prescribed.

The first improvement in the case was noted soon after giving the twelve grains of quinine daily for a week. During the summer and autumn of 1900 there was a slow but manifest improvement. The remains of the old bullæ gradually disappeared and fewer new ones were formed.

At the present time, February 1, 1901, there has been no outbreak for several weeks; hardly a single bullous crust remains on the body. The patient is able to attend to all her ordinary household duties and goes wherever she wishes. Although not quite as strong as two years ago, she is free from pain and the pemphigoid itching and burning.

During the progress of the case there were three subacute attacks of gastro-enteritis, each attack lasting a week. It is possible that these attacks were induced by the long-continued large doses of arsenic.

Nearly two years have elapsed since the initial symptoms of pemphigus appeared in this case. It is the belief of the writer that to a dogged persistency in the use of internal and external remedies is due its successful outcome.

How long the present happy condition of the patient will continue is problematical. Chronic pemphigus sometimes occupies many years and has many remissions and exacerbations. Time only can determine whether this patient has been fully and for life delivered from the distressing remedy-resisting disease, chronic pemphigus.

BIBLIOGRAPHY.

1. Disaeses of the skin. Hebra. Vol. 2, p. 364.
2. Journal of Cutaneous and Genito-Urinary Diseases, 1888, p. 121.
3. London Lancet, 1890, Vol. 1, p. 1190.
4. Journal of Cutaneous and Genito-Urinary Diseases, 1893, p. 498.
5. London Lancet, 1858, Vol. 1, p. 314.
6. London Lancet. 1889, Vol. 2, p 1119.
7. Cazenave on the Skin, 2d. American edition, 1852, p. 120.
8. London Lancet, 1870, Vol. 1, p. 897.
9. New York Medical Journal, July 3, 1897, p. 1.
10. London Lancet, 1888, Vol 2, p. 527.
11. London Lancet, 1885, Vol. 2, p. 996.
12. Journal of Cutaneous and Genito-Urinary Diseases, 1889, p. 453.
13. Diseases of the Skin, 4th edition, Hyde, p. 395.
14. Diseases of the Skin, 4th edition, Hyde, p. 395.
15. London Lancet, 1889, Vol. 1, p. 531.
16. Journal of Cutaneous and Genito-Urinary Diseases, 1891, pp. 332 and 423.
17. London Lancet, 1873, Vol. 2, p. 456.

18. London Lancet, 1873, Vol. 1, p. 875.
19. Braithwaite, 1885, Vol. 1, p. 62.
20. Journal of Cutaneous and Genito-Urinary Diseases, 1888, p. 123.
21. London Lancet, 1890, Vol. 1, p. 1190.
22. Journal of Cutaneous and Genito-Urinary Diseases, 1885, p. 220.
23. Diseases of the Skin, 4th edition, Hyde, p. 391.
24. Diseases of the Skin, 3rd. edition, Duhring, p. 252.
25. Diseases of the Skin, 3rd. edition, Duhring, p. 252.
26. Journal of Cutaneous and Genito-Urinary Diseases, 1888, p. 121.
27. Journal of Cutaneous and Genito-Urinary Diseases, 1888, p. 121.
28. Manual of Dermatology, Robinson, p. 238.
29. Transactions of American Dermatological Association, 1896, p. 65.
30. Diseases of the Skin, Hebra, Vol. 2, p. 375.
31. Diseases of the Skin, Hebra, p. 376.
32. Diseases of the Skin, 3rd. edition, Duhring, p. 252.
33. Manual of Dermatology, Robinson, p. 238.
34. Diseases of the Skin, 4th. edition, Hyde, p. 395.
35. London Lancet, 1881, Vol. 1, p. 53.
36. Cutaneous Medicine and Diseases, Wilson, p. 169.
37. London Lancet, 1887, Vol. 2, p. 858.
38. London Lancet, 1879, Vol. 2, p. 940.
39. London Lancet, 1889, Vol. 2, p. 791.
40. London Lancet, 1888, Vol. 1, p. 1131.
41. Journal of Cutaneous and Genito-Urinary Diseases, 1892, p. 483.
42. Cutaneous Medicine and Diseases, Wilson, p. 168.
43. Nelligan on Diseases of the Skin, 4th. American edition, p. 97.
44. Handbook of Diseases of the Skin, Ziemssen, p. 263.
45. Handbook of Diseases of the Skin, Ziemssen, p. 263.
46. London Lancet, 1889, Vol. 2, p. 807.
47. Archives of Dermatology, 1880, p. 77.
48. Archives of Dermatology, 1878, p. 83.
49. Archives of Dermatology, 1874-5, p. 154.
50. Archives of Dermatology, 1874-5, p 154.
51. Handbook of Diseases of the Skin, Ziemssen, p. 264, foot note.
52 Archives of Dermatology, 1879, p. 285.
53. London Lancet, 1890, Vol. 1, p. 850.
54. London Lancet, 1886, Vol. 1, p. 140.
55. British Medical Journal, 1889, Vol. 2, p. 1150.
56. Skin Diseases, Tilbury Fox, p. 212.
57. Diseases of the Skin, Hebra, Vol. 2, p. 384.
58. Diseases of the Skin, 3rd. edition, Duhring, p. 252 and Manual of Dermatology, Robinson, p. 238.
59. London Lancet, 1890, Vol. 1, p. 1190.
60. London Lancet, 1898, Vol. 2, p. 1397.
61. New York Medical Journal, 1898, Vol. 67, p. 175.
62. Manual of Dermatology, Robinson, p. 238.
63. Diseases of the Skin, 4th. edition, Hyde, p. 395.
64. Diseases of the Skin, 4th. edition, Hyde, p. 397.
65. Medical Diagnosis, Vierordt, p 581.
66. Diseases of the Skin, 4th. edition, Hyde, p. 396.
67. Diseases of the Skin, 4th. edition, Hyde, p. 397.
68. Handbook of Diseases of the Skin, Ziemssen, p. 266.
69. Handbook of Diseases of the Skin, Ziemssen, p. 266.
70. London Lancet, 1885, Vol. 1, p. 981.
71. Diseases of the Skin, 4th. edition, Hyde, p. 397.
72. Diseases of the Skin, 4th. edition, Hyde, p. 397.
73. Diseases of the Skin, 4th. edition, Hyde, p. 397.
74. London Lancet, 1885, Vol. 1, p. 981.
75. British and Foreign Medico-Chirurgical Review, 1876, Vol. 2, p. 479.
76. British and Foreign Medico-Chirurgical Review, 1876, Vol. 2, p. 480.
77. Archives of Dermatology, 1877, p. 157.
78. London Lancet, 1888, Vol. 1, p. 1131.

79. Handbook of Diseases of the Skin, Ziemssen, p. 263.
80. Handbook of Diseases of the Skin, Ziemssen, p. 263.
81. Skin Diseases, Tilbury Fox, p. 213.
82. Diseases of the Skin, 3rd. edition, Duhring, p. 253.
83. Neumann's Handbook of Skin Diseases, Bulkley's American edition, p. 190.
84. Nelligan on Diseases of the Skin, p. 100.
85. Handbook of Diseases of the Skin, Ziemssen, p. 266.
86. Skin Diseases, Tilbury Fox, p. 211.
87. Manual of Dermatology. Robinson, p. 240.
88. Diseases of the Skin, Hebra, Vol. 2, p. 372.
89. Diseases of the Skin, 4th. edition. Hyde, p. 396.
90. Skin Diseases, Tilbury Fox, p. 215.
91. Manual of Dermatology, Robinson, p. 240.
92. Diseases of the Skin, 3rd edition, Duhring, p. 255.
93. Journal of Cutaneous and Genito-Urinary Diseases, 1890, p. 471.
94. Diseases of the Skin, 4th. edition, p. 399.
95. Therapeutics of the Skin, Piffard, 1881, p. 236.
96. Skin Diseases, Tilbury Fox, p. 215.
97. Diseases of the Skin, 3rd. edition, Duhring, p. 266.
98. Journal of Cutaneous and Genito-Urinary Diseases, 1891, pp. 332 and 423.
99. Neumann's Hand Book of Skin Diseases, Bulkley's American edition, p. 194.
100. British and Foreign Medico-Chirurgical Review, 1872, Vol. 49, p. 535.
101. Diseases of the Skin, Hebra, p. 396.
102. Braithwaite, 1889, Vol 1, p. 120.
103. Diseases of the Skin, 4th. edition, Hyde, p. 400.
104. Diseases of the Skin, Hebra, Vol. 2, p. 397.

118 PLYMOUTH AVENUE.

